# A Rare Case of Fungal Osteomyelitis of the Distal Tibia in a Pediatric Patient

**DOI:** 10.7759/cureus.54648

**Published:** 2024-02-21

**Authors:** Syed Faisal Afaque, Udit Agrawal, Dilip K Shankhwar, Suresh Chand, Vikas Verma

**Affiliations:** 1 Paediatric Orthopaedics, King George's Medical University, Lucknow, IND; 2 Orthopaedics, Jawaharlal Nehru Medical College, Aligarh, IND

**Keywords:** distal tibia, rare case report, paediatric, osteomyelitis, fungal

## Abstract

*Staphylococcus aureus* infection is the most common cause of osteomyelitis. Over 100,000 fungal species have been described; only 150 are pathogenic to humans. These opportunistic infections frequently enter the body due to a decrease in host defense or through an invasive gateway, such as a dental extraction or skin discontinuity due to trauma. Symptoms and radiological examination often mimic those of other etiologies, which can lead to substantial delays in treatment. Our case is a 13-year-old healthy boy with no history of immune incompetency who presented to us with complaints of pain and swelling over his left ankle and leg with an on-and-off history of fever for 15 days. Based on his history and examination, he is diagnosed as having sub-acute osteomyelitis of the distal tibia with septic arthritis. The bacterial culture has no growth; however, the potassium hydroxide mount came positive for fungal elements having hyphae and pseudohyphae, and the fungal culture came positive for *Candida*. Management of fungal infections is challenging as they have infrequent involvement in bones. Fungal osteomyelitis is considered a rare entity in the literature, and the current case is studied for the management and diagnosis of a rare variant of osteomyelitis in the pediatric population. The treatment guidelines vary based on the identified organism and the duration of treatment. Debridement of fungal osteomyelitis or septic arthritis includes removing sinus tracts, evaluation for squamous cell carcinoma, bony and soft-tissue debridement, and antibiotic or antifungal bead placement. The spectrum of osteomyelitis ranges from *Staphylococcus aureus* organisms to tumors; therefore, it is necessary to investigate every spectrum of the disease, and fungal infections should be considered differential even though they are a rare entity. Early diagnosis, surgical debridement, and proper antifungal treatment based on fungal species lead to better clinical outcomes and results.

## Introduction

Osteomyelitis is an infection of the bone and bone marrow most commonly caused by a *Staphylococcus aureus* infection [1]. It can be acute or chronic inflammatory, involving the bone and its structures secondary to infection with pyogenic organisms, including bacteria, viruses, fungi, and mycobacterial species [2]. *Staphylococcus aureus* is the causative pathogen in 30% to 60% of human cases [3], and staphylococci collectively cause approximately 75% of cases [4]. It is most commonly spread by a hematogenous route followed by a fracture or surgery.

Over 100,000 fungal species have been described; only 150 are pathogenic to humans [5]. The common fungal pathogens in septic arthritis and osteomyelitis are *Aspergillus* and *Candida* species [6]. These opportunistic infections frequently enter the body due to a decrease in host defense, poor nutritional status, or through an invasive gateway, such as skin discontinuity due to trauma [7]. Symptoms and radiological examination often mimic those of other etiologies, which can lead to substantial delays in treatment. Aspergillus infection is seen in most patients and commonly involves the vertebral bone [8]. Fungal osteomyelitis is rarely seen in the pediatric population and often remains misdiagnosed. As only minimal literature is available, we are submitting a case report of fungal osteomyelitis in a pediatric patient.

## Case presentation

**Figure 1:**
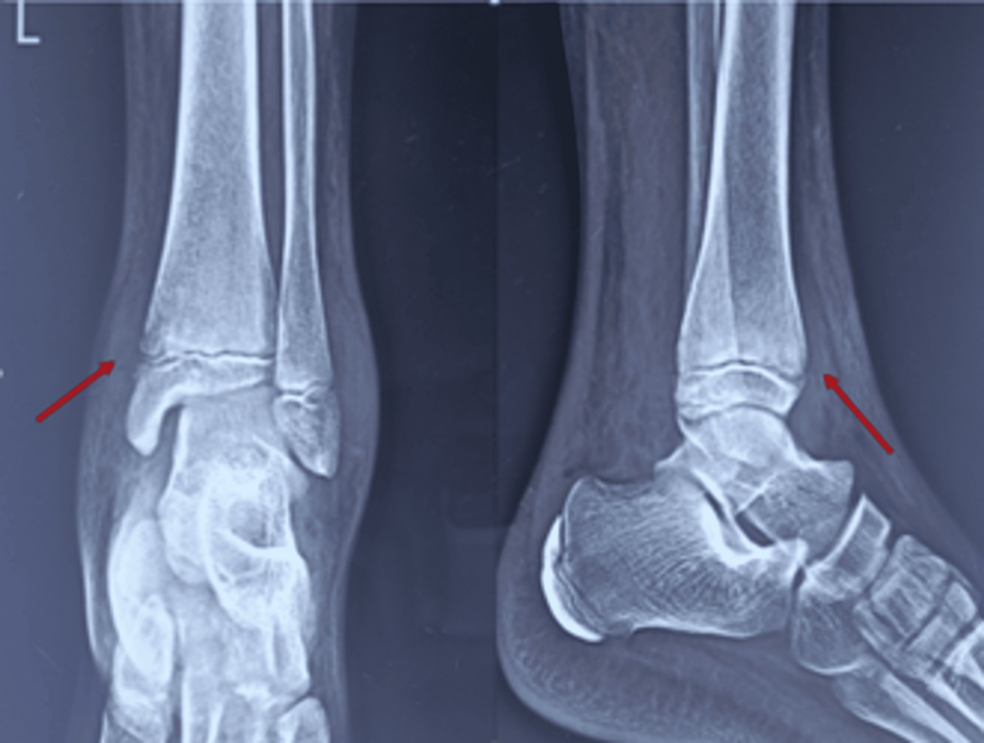
Plane radiograph showing soft tissue outline with swelling and regional osteopenia over the anteromedial aspect of the distal tibia with irregularity of the physeal margin (arrow)

Our case is a 13-year-old healthy boy with no history of immune incompetency who presented to us with complaints of pain and swelling over his left ankle and leg with an on-and-off history of fever for 15 days. In routine hematological investigations, there is leukocytosis with raised CRP and ESR. In-plane radiographs show a soft tissue outline with swelling over the anteromedial aspect of the distal tibia, irregularity of the physeal margin, and osteopenia with no periosteal reaction (Figure 1).

**Figure 2:**
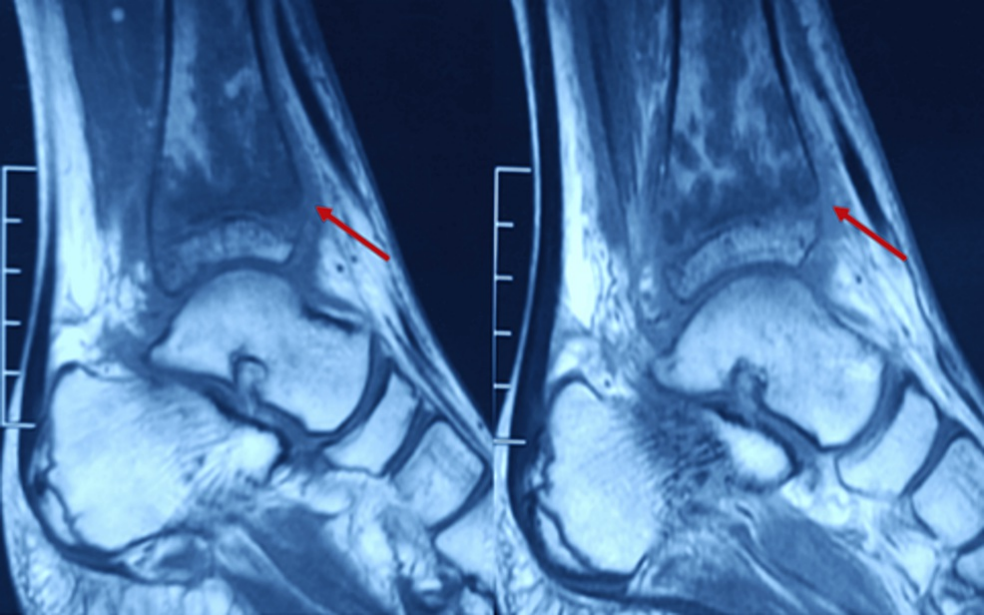
Sagittal MRI section of the distal tibia showing osteomyelitis changes (arrow)

**Figure 3:**
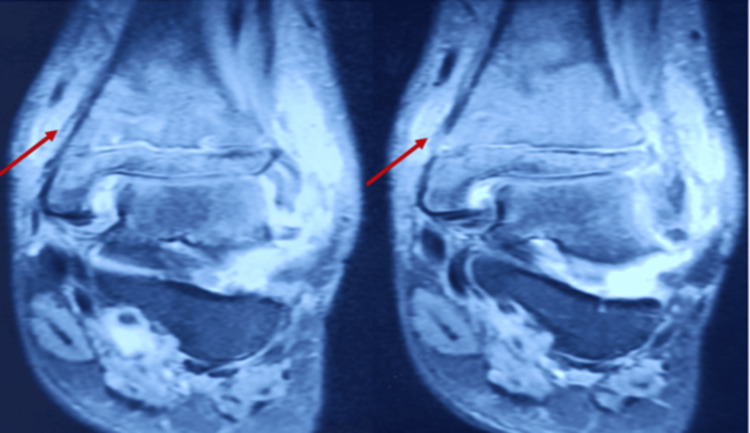
T2 coronal MRI section showing osteomyelitis changes in the distal tibia (arrow)

MRI suggests nodular synovial thickening with effusion in the ankle joint with serpiginous intramedullary bone infracts (Figures 2-3). Based on his history and examination, he is diagnosed with osteomyelitis of the distal tibia and septic arthritis.

**Figure 4:**
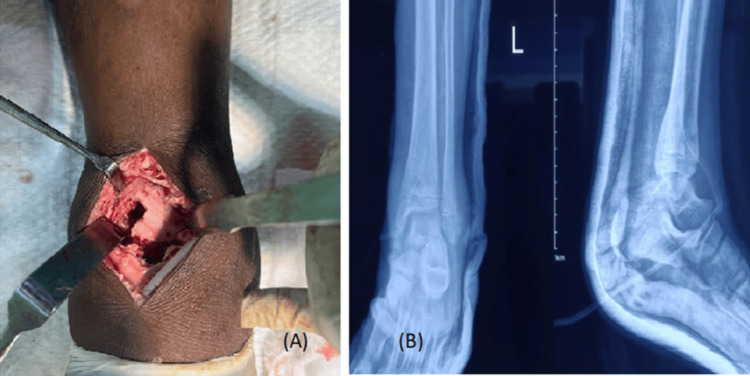
(A) Distal tibial cortical window and (B) postoperative radiograph

In the preoperative period, no antibiotic was given to the patient. After proper anesthesia, the patient was positioned supine on the operation table, and a vertical anterior incision was given midway between both the malleolus. A superficial dissection is done by incising the deep fascia and extensor retinaculum. The deep plane is created between the extensor digitorum longus tendon and the extensor hallucis longus. The neurovascular bundle was identified and retracted medially, along with the extensor hallucis longus and the extensor digitorum longus tendon retracted laterally. The joint capsule was incised, and the collection was non-purulent when the distal tibial cortical window (Figure 4A) was made. The aspirates and soft tissue samples were sent for Gram stain, aerobic and anaerobic culture sensitivity, cartridge-based nucleic acid amplification test, acid-fast bacteria stain, potassium hydroxide (KOH) mount, and histopathological examination. The wound was closed in layers, a drain was put in, the postoperative dressing was done on postoperative days 2 and 5, and sutures were removed after 14 days of the initial surgery. A postoperative radiograph was taken (Figure 4B).

Postoperatively, the patient prophylactically started on ceftriaxone and vancomycin injectables, and the dose was adjusted according to the weight. There was a decrease in total leucocyte counts, ESR, and CRP trends. The bacterial culture shows no growth; however, the KOH mount came positive for fungal elements having hyphae and pseudo-hyphae, and the fungal culture came positive for *Candida* and sensitive to fluconazole. The patient was put on intravenous fluconazole 200 mg (12 mg/kg/day) STAT dose, followed by 120 mg (6 mg/kg/dose) given once a day for a total of 28 days. The swelling completely subsided in 18 days, with no residual ankle stiffness.

## Discussion

Management of fungal infections is challenging as they have infrequent involvement in bones. Fungal osteomyelitis is considered a rare entity in the literature, and the current case is studied for the management and diagnosis of a rare variant of osteomyelitis in the pediatric population. The clinical presentation of bacterial and fungal osteomyelitis was similar [9]. Fungal osteomyelitis is an uncommon form of osteomyelitis that often occurs concurrently with septic arthritis [10].

The treatment guidelines vary based on the identified organism and the duration of treatment. Debridement of fungal osteomyelitis or septic arthritis includes removing sinus tracts, evaluation for squamous cell carcinoma, bony and soft-tissue debridement, and antibiotic or antifungal bead placement. In fungal septic arthritis, the joint is thoroughly irrigated and debrided surgically. However, surgical procedures are not necessarily required to manage some fungi, such as *Cryptococcus*, if the disease burden is mild [6]. Fluconazole inhibits the demethylation of C-14 sterols, accumulates abnormal methyl sterols, increases cellular permeability, and has inhibitory action against fungal cells [11]. In their case report, Pan et al. also preferred fluconazole to treat candidal osteomyelitis in pediatric patients, which has shown promising outcomes [12].

## Conclusions

The spectrum of osteomyelitis ranges from *Staphylococcus aureus* organisms to tumors; therefore, it is necessary to investigate every spectrum of the disease. Fungal infection should be considered a differential, even though it is a rare entity. Early diagnosis, surgical debridement, and proper antifungal treatment based on fungal species lead to better clinical outcomes and results.

## References

[1] Hofstee MI, Muthukrishnan G, Atkins GJ (2020). Current concepts of osteomyelitis: from pathologic mechanisms to advanced research methods. Am J Pathol.

[2] Schmitt SK (2017). Musculoskeletal infections: meeting the challenge. Infect Dis Clin North Am.

[3] Tong SY, Davis JS, Eichenberger E, Holland TL, Fowler VG Jr (2015). Staphylococcus aureus infections: epidemiology, pathophysiology, clinical manifestations, and management. Clin Microbiol Rev.

[4] Walter G, Kemmerer M, Kappler C, Hoffmann R (2012). Treatment algorithms for chronic osteomyelitis. Dtsch Arztebl Int.

[5] Bongomin F, Gago S, Oladele RO, Denning DW (2017). Global and multi-national prevalence of fungal diseases-estimate precision. J Fungi (Basel).

[6] Bariteau JT, Waryasz GR, McDonnell M, Fischer SA, Hayda RA, Born CT (2014). Fungal osteomyelitis and septic arthritis. J Am Acad Orthop Surg.

[7] Bhansali A, Bhadada S, Sharma A (2004). Presentation and outcome of rhino-orbital-cerebral mucormycosis in patients with diabetes. Postgrad Med J.

[8] Asperges E, Albi G, Truffelli F (2023). Fungal osteomyelitis: a systematic review of reported cases. Microorganisms.

[9] Urs AB, Singh H, Mohanty S, Sharma P (2016). Fungal osteomyelitis of maxillofacial bones: a rare presentation. J Oral Maxillofac Pathol.

[10] Weerakkody Y (2023). Fungal Osteomyelitis. https://radiopaedia.org/articles/fungal-osteomyelitis.

[11] Fleming L, Ng A, Paden M, Stone P, Kruse D (2012). Fungal osteomyelitis of calcaneus due to Candida albicans: a case report. J Foot Ankle Surg.

[12] Pan N, Herzog R, Blanco JS (2013). Candida albicans osteomyelitis in an infant: a case report and literature review. J Pediatr Orthop B.

